# Functional characterization and transcriptional repression by *Lacticaseibacillus paracasei* DinJ-YafQ

**DOI:** 10.1007/s00253-022-12195-4

**Published:** 2022-10-04

**Authors:** Aleksandra Anna Bonini, Stefano Maggi, Giulia Mori, Dario Carnuccio, Danila Delfino, Davide Cavazzini, Alberto Ferrari, Alessia Levante, Yoshihiro Yamaguchi, Claudio Rivetti, Claudia Folli

**Affiliations:** 1 Department of Chemistry, Life Sciences and Environmental Sustainability, University of Parma, 43124 Parma, Italy; 2 Department of Food and Drug, University of Parma, 43124 Parma, Italy; 3 Department of Biology, Osaka Metropolitan University, Sugimoto, Sumiyoshi-ku, Osaka, 558-8585 Japan

**Keywords:** Toxin-antitoxin, DinJ-YafQ, Oligomerization, Transcription repression, Atomic force microscopy, GFP

## Abstract

**Abstract:**

DinJ-YafQ is a bacterial type II TA system formed by the toxin RNase YafQ and the antitoxin protein DinJ. The activity of YafQ and DinJ has been rigorously studied in *Escherichia coli*, but little has been reported about orthologous systems identified in different microorganisms. In this work, we report an *in vitro* and *in vivo* functional characterization of YafQ and DinJ identified in two different strains of *Lacticaseibacillus paracasei* and isolated as recombinant proteins. While DinJ is identical in both strains, the two YafQ orthologs differ only for the D72G substitution in the catalytic site. Both YafQ orthologs digest ribosomal RNA, albeit with different catalytic efficiencies, and their RNase activity is neutralized by DinJ. We further show that DinJ alone or in complex with YafQ can bind cooperatively to a 28-nt inverted repeat overlapping the −35 element of the TA operon promoter. Atomic force microscopy imaging of DinJ-YafQ in complex with DNA harboring the cognate site reveals the formation of different oligomeric states that prevent the binding of RNA polymerase to the promoter. A single amino acid substitution (R13A) within the RHH DNA-binding motif of DinJ is sufficient to abolish DinJ and DinJ-YafQ DNA binding in vitro. In vivo experiments confirm the negative regulation of the TA promoter by DinJ and DinJ-YafQ and unveil an unexpected high expression-related toxicity of the *gfp* reporter gene. A model for the binding of two YafQ-(DinJ)_2_-YafQ tetramers to the promoter inverted repeat showing the absence of protein-protein steric clash is also presented.

**Key points:**

• *The RNase activity of L. paracasei YafQ toxin is neutralized by DinJ antitoxin.*

• *DinJ and DinJ-YafQ bind to an inverted repeat to repress their own promoter.*

• *The R13A mutation of DinJ abolishes DNA binding of both DinJ and DinJ-YafQ.*

**Supplementary Information:**

The online version contains supplementary material available at 10.1007/s00253-022-12195-4.

## Introduction

Toxin-antitoxin (TA) systems are small genetic elements widespread in bacterial chromosomes and plasmids, which typically encode for a noxious protein and for an antitoxin protein or a non-coding RNA capable of neutralizing toxin activity (Yamaguchi et al. 2011). TA systems are grouped in eight distinct classes based on the moiety of the antitoxin and the mechanism of action (Song and Wood 2020). In type II TA systems, which are relevant to this study, both the toxin and the antitoxin are proteins that form a stable inactive complex. Under stress-conditions, such as nutrient starvation, oxidative stress, and antibiotic challenge, degradation of the antitoxin or de novo synthesis of toxin causes a non-stoichiometric balance of the two polypeptides granting the toxin to inhibit cell growth (Song and Wood 2020; Kamruzzaman et al. 2021). Thus, a fine regulation of the level of expression of these two proteins represents a key feature of the toxin-antitoxin homeostasis. In most cases, TA genes are organized as operons, usually with the antitoxin ORF located upstream of the toxin ORF (Gerdes et al. 2005). Besides neutralizing the toxin activity, the antitoxin and the toxin-antitoxin complex can act as transcriptional repressors of the TA operon by binding to an operator site within the promoter region (Fraikin et al. 2020). The ability to form TA complexes with different stoichiometries underlies the phenomena known as conditional cooperativity which can buffer the TA system against fluctuations in the expression of the toxin and the antitoxin. An antitoxin dimer binds the TA promoter to repress transcription; binding of the toxin to the antitoxin allows cooperative recruitment at the operator site, strengthening the repression. However, when the toxin/antitoxin ratio exceeds a certain threshold, the TA-operator complex is destabilized resulting in derepression of the TA operon (Cataudella et al. 2012; Chan et al. 2016; Harms et al. 2018; Fraikin et al. 2020). In addition to transcriptional regulation, the majority of type II toxins can also function as translation interfering elements by means of their ribosome-dependent (RelE, YoeB, YafQ, YafO, HigB) or ribosome-independent (MqsR, MazF) endoribonuclease activity (Han and Lee 2020).

The DinJ-YafQ TA system has been first identified and functionally characterized in *Escherichia coli* (Motiejūnaite et al. 2007). The YafQ toxin is an endoribonuclease belonging to the RelE/YoeB toxin family that not only binds to the ribosome and specifically cleaves the translating mRNA in vivo (Prysak et al. 2009), but it also shows a significant ribosome-independent RNase activity in vitro (Liang et al. 2014). The crystallographic structure of DinJ-YafQ has revealed a tetrameric organization of the complex in which two DinJ-YafQ dimers are associated through the interaction of DinJ N-terminal regions (Liang et al. 2014; Ruangprasert et al. 2014). The YafQ structure shows a concave surface containing a sulfate anion (arising from the crystallization solution) near the proposed catalytic residues, which most probably represents the site of interaction with the negative phosphate of the cleaved RNA backbone. Toxin inactivation is mediated by a DinJ linker region that, through salt bridge and π-stacking interactions, obstructs the YafQ active site (Ruangprasert et al. 2014, 2017). Repression of the TA operon, on the other hand, is mediated by the formation of a canonical RHH DNA-binding motif upon DinJ dimerization through its N-terminal region (Liang et al. 2014; Ruangprasert et al. 2014). In vitro studies have demonstrated that both DinJ and DinJ-YafQ specifically bind to palindromic sequences inside the *dinJ-yafQ* promoter (Armalytė et al. 2012; Liang et al. 2014; Ruangprasert et al. 2014), while in vivo experiments provided evidence for the DinJ and DinJ-YafQ mediated repression of the *dinj-yafQ* promoter (Ruangprasert et al., 2014).

Recently, we identified DinJ-YafQ TA systems in different *Lacticaseibacillus* strains isolated from dairy products and evaluated their expression in stress conditions related to food production processes (Levante et al. 2019; Ferrari et al. 2019). The identified YafQ orthologs are distributed among *Lacticaseibacillus rhamnosus* and *L. paracasei* strains maintaining high intraspecies sequence identity and synteny with DinJ, in spite of different functional activities (Ferrari et al., 2019). In particular, YafQ from *L. paracasei* 4366 (YafQ_pa4366) caused a significant growth inhibition when expressed in *E. coli*, while a very limited effect was observed in the case of YafQ from *L. paracasei* 2333 (YafQ_pa2333). Interestingly, YafQ_pa4366 and YafQ_pa2333 differ only for a single D72G substitution mapped in the proposed active site. Conversely, the DinJ antitoxin has the same amino acid sequence in both strains (Ferrari et al. 2019). In this work, we report results of in vitro and in vivo experiments aimed at characterizing the YafQ enzymatic activity and the role of DinJ/YafQ in the transcriptional regulation of the *dinJ-yafQ* promoter using recombinant proteins identified in the strains 4366 and 2333 of *L. paracasei*.

## Materials and methods

### Gene cloning for protein overexpression

The strains 4366 and 2333 of *L. paracasei*, isolated from dairy matrices, are part of the University of Parma Culture Collection (UPCC). The *yafQ* 2333 and 4366 ORFs (GenBank accession MK544943 and MK544944) were amplified from the total DNA extracted from *L. paracasei* 2333 and 4366 strains as described in Levante et al. (2021). The region encoding for DinJ, which has identical amino acid sequence in both strains 4366 and 2333 (Ferrari et al. 2019), was amplified from total DNA of *L. paracasei* 4366 (Tables S1 and S2). All the amplified fragments were first cloned into pGEM-T easy vector (Promega) and subsequently cloned into the *Nde*I/*Bam*HI restriction sites of the inducible expression vectors pET11b or pET28b to generate two versions of both DinJ and YafQ: one fused with an N-terminal hexa-His tag and another one without any fusion tag (Table S1). The plasmid pET28b-*dinJ*R13A encoding the DinJ variant R13A was obtained by site-directed mutagenesis using plasmid pET28-*dinJ* as template, the high-fidelity Pfu Ultra II Fusion HS DNA polymerase (Stratagene) and mutagenic primers complementary to the opposite DNA strands (Table S2). The DNA product of the reaction was treated with *Dpn*I enzyme (New England Biolabs) to digest the parental DNA template and used to transform *E. coli* XL1 Blue cells. All constructs were verified by DNA sequencing. Finally, to produce YafQ of the two *L. paracasei* strains, *E. coli* BL21 (DE3) cells were co-transformed with pET11b-*dinJ* and pET28b-*yafQ*_pa4366 or pET28b-*yafQ*_pa2333. To obtain wt DinJ or DinJR13A, *E. coli* BL21 (DE3) cells were transformed with pET28b-*dinJ* or pET28b-*dinJ*R13A. To obtain DinJ-YafQ complexes, *E. coli* BL21 (DE3) cells were co-transformed with pET28b-*dinJ* or pET28b-*dinJ*R13A and pET11b-*yafQ*_pa4633 or pET11b-*yafQ*_pa2333 (Table S1).

### Recombinant protein expression and purification

Recombinant cells were grown in Luria-Bertani (LB) medium (10 g/L tryptone, 10 g/L NaCl, and 5 g/L yeast extract), supplemented with 50 μg/mL kanamycin for pET28b or 100 μg/mL ampicillin for pET11b, until OD_600_ reached 0.5–0.8. Protein expression was induced with 1 mM isopropyl β-D-thiogalactoside (IPTG) at 20 °C for 16 h. Cells were harvested by centrifugation at 4500 × g for 15 min at 4 °C. Samples were analyzed for expression on 15% SDS-PAGE and visualized by Coomassie staining. The pellet from a 1-L culture was resuspended in 40 mL lysis buffer (50 mM Tris-HCl, pH 7.5, 250 mM KCl, 5 mM MgCl_2_, 15 mM imidazole), sonicated on ice and then centrifuged at 13,300 × g for 30 min. All the recombinant proteins were purified from culture supernatants.

DinJ and the DinJ-YafQ complexes were purified by affinity chromatography on a HisTrap FF crude column connected to an ÄKTA Pure FPLC System (GE Healthcare). After loading supernatant, the column was washed with 2 M NaCl to remove nucleic acid contaminants. Protein fractions were eluted with elution buffer (50 mM Tris-HCl, pH 7.5, 250 mM KCl, 5 mM MgCl_2_, 500 mM imidazole), pooled, and exchanged with the same buffer without imidazole using Hi-Trap desalting columns (GE Healthcare). Protein quality was checked by SDS-PAGE, and concentration was estimated by measuring absorbance at 280 nm using the protein extinction coefficient generated by ProtParam (https://web.expasy.org/protparam/; for DinJ: *ε*_280_ = 6990 M^−1^·cm^−1^; for DinJ-YafQ: *ε*_280_ = 29450 M^−1^·cm^−1^). Aliquots of purified protein were snap-frozen in liquid nitrogen and stored at −80 °C. YafQ was purified by affinity chromatography following dissociation from the DinJ-YafQ complex in denaturing conditions. In detail, the supernatant containing the DinJ-YafQ complex was loaded on a His-Trap FF crude column connected to an ÄKTA Pure FPLC System (GE Healthcare). The column was washed with denaturing buffer (50 mM Tris-HCl, pH 7.5, 250 mM KCl, 5 mM MgCl_2_, 15 mM imidazole, 6 M guanidine-HCl) to remove DinJ. Elution was performed with a denaturing elution buffer (50 mM Tris-HCl, pH 7.5, 250 mM KCl, 5 mM MgCl_2_, 500 mM imidazole, 6 M guanidine-HCl) and the eluted protein was exchanged into the same buffer without imidazole and guanidine-HCl using Hi-Trap desalting columns (GE Healthcare). The final protein concentration was estimated by measuring absorbance at 280 nm (*ε*_280_ = 22460 M^−1^·cm^−1^). Aliquots of purified protein were snap-frozen in liquid nitrogen and stored at −80 °C.

### RNase activity assays

RNase activity was measured by disappearance of ribosomal RNA bands in 1% agarose gel electrophoresis stained with Midori green (NIPPON Genetics). The 10-μL reaction mixes containing 170 ng of RNA in 10 mM Tris-HCl, pH 7.5, and increasing concentrations of YafQ or DinJ-YafQ complex were prepared at room temperature and incubated at 37 °C for 15 min. The reactions were quenched by adding 2 μL of loading dye (10 mM Tris-HCl pH 7.6, 0.03% bromophenol blue, 0.03% xylene cyanol, 60% glycerol, 60 mM EDTA). After electrophoresis, the gel image was recorded using a ChemiDoc MP imager (Bio-Rad).

### Gel mobility shift assays

A 194-bp DNA fragment harboring the promoter region of the *dinJ-yafQ* operon was PCR-amplified under standard conditions from *L. paracasei* 4366 total DNA by using primers labeled with DY682 fluorophore (Table S2). The EMSA reactions (total volume 10 μL) containing 2 nM DNA, 128 ng of salmon sperm DNA, and increasing concentrations of DinJ or DinJ-YafQ complex in buffer 50 mM Tris-HCl, pH 7.5, 50 mM KCl, 5% glycerol were prepared at room temperature and incubated for 10 min. The samples were electrophoresed on a 6% native polyacrylamide gel in TBE buffer (45 mM Tris, 45 mM boric acid, 1 mM EDTA, pH 8.3) for 70 min at 90 V at room temperature using a Mini-Protean apparatus (Bio-Rad). The gel image was recorded using a ChemiDoc MP imager and unbound DNA bands were quantified by densitometric analysis using Image Lab software (Bio-Rad). The fraction of bound DNA, determined with respect to the lane without protein added, was plotted as a function of protein concentrations and fitted by using a Hill equation: $\mathrm{fraction}\;\mathrm{of}\;\mathrm{bound}\;\mathrm{DNA}=1/(1+{(K_{\mathrm{Dapp}}/\left[P\right])}^n)$, where *K*_Dapp_ is the apparent dissociation constant, [*P*] the DinJ or DinJ-YafQ protein concentration, and *n* the Hill constant.

### Atomic force microscopy and image analysis

Protein-DNA complexes were assembled using 20 nM DNA and 100 nM DinJ-YafQ in buffer 4 mM HEPES pH 7.4, 50 mM KCl, and 2 mM MgCl_2_. The reactions were incubated at 25 °C for 30 min prior to the addition of glutaraldehyde to a final concentration of 0.1%, further incubated for 10 min and quenched by adding Tris-HCl pH 8 to a final concentration of 2 mM. The reactions were diluted 10-fold in deposition buffer (4 mM HEPES pH 7.4, 10 mM NaCl, 2 mM MgCl_2_), and a 20 µL drop was deposited onto freshly cleaved mica for 2 min before the surface was rinsed with Milli-Q water and dried by nitrogen. AFM images (512 × 512 pixels with a scan size of 2 µm) were collected in air with a Nanoscope IIIA microscope (Digital Instruments, Santa Barbara, CA, USA) operating in tapping mode and equipped with the E scanner. Commercial silicon cantilevers (MikroMasch, Tallinn, Estonia) with a nominal tip radius of 5 nm were used.

The position of DinJ-YafQ bound on the DNA template was determined by measuring the contour length of the two DNA arms as described in Doniselli et al. (2015). The volume of DinJ-YafQ/DNA complexes was measured using an ad hoc procedure written in Matlab (Mathworks, Natick, MA, USA) as follows. The Nanoscope image pixel values were converted to height (nm), and the image was shifted to zero by subtracting the minimum value. Each complex was outlined with an ellipse to obtain the image mask of the complex. The area and the height of the pixels within the mask were used to determine the volume of the complex. The volume of the protein moiety was obtained by subtracting the volume of the DNA moiety measured in the proximity of the complex. The reference background value was determined in a 6 × 6-pixel region near the complex (Fig. S1).

The volume/MW calibration curve used to infer the stoichiometry of DinJ-YafQ/DNA complexes was obtained using four globular proteins of known molecular mass (Fig. S1). Protein samples were diluted in deposition buffer (4 mM HEPES pH 7.4, 10 mM NaCl, 2 mM MgCl_2_) to reach a concentration of 20 nM and deposited onto freshly cleaved mica. AFM images of the rinsed and dried samples were collected with a scan size of 2 µm. Protein volume was measured using the “Zero basis” volume algorithm of the Gwyddion software v2.60 (Nečas and Klapetek 2012).

### DNA binding competition

For the competition with RNA polymerase, the fluorescently labeled 194 bp DNA fragment employed for the EMSA assays was used. The 10-μL reaction contained 2 nM DNA, 128 ng of salmon sperm DNA, and increasing concentrations of DinJ-YafQ in 20 mM Tris-HCl, pH 7.9, 50 mM KCl, 5 mM MgCl_2_ and 5% glycerol. The reaction was incubated at room temperature for 10 min; afterwards, 200 nM *E. coli* RNAP holoenzyme (New England BioLabs) was added and the reaction was further incubated at 37 °C for 30 min. The samples were loaded on a 2% agarose gel in TB buffer (45 mM Tris, 45 mM boric acid, pH 8.3) and electrophoresed for 50 min at 20 V/cm at room temperature. In the control experiment, the DNA was replaced by a fluorescently labeled 196 bp DNA fragment harboring the lambda PR promoter. Gel images were recorded by using a ChemiDoc MP imager.

### In vivo transcriptional repression assays

The promoter region of the *dinJ-yafQ* operon was amplified by PCR from the total DNA extracted from *L. paracasei* 4366, and the *gfp* ORF was amplified by PCR from pET28CpoI-*gfp*mut2 (Abbruzzetti et al. 2005). The two amplified overlapping DNA fragments were fused by PCR, and the product was cloned into the BglII/HindIII restriction sites of pET28b vector, pET28b-*dinJ* and pET28b-*dinJ*R13A (primers are reported in Table S2). Constructs were verified by DNA sequencing and transformed or co-transformed with pET11b-*yafQ*_pa2333 into C41(DE3) pLysS *E. coli* strain by electroporation. Single colonies picked from the transformation plates were restreaked on fresh LB-agar plates with or without 0.5 mM IPTG and incubated overnight at 37 °C. Plates were scanned with a ChemiDoc MP imager. Single colonies were also grown in LB medium supplemented with 50 μg/mL kanamycin for pET28b and/or 100 μg/mL ampicillin for pET11b, overnight at 37 °C. Cultures were diluted to an OD_600_ of about 0.10 in fresh LB medium with or without 0.5 mM IPTG and grown at 37 °C. One milliliter of culture collected in the mid-log phase after 2 h of growth was pelleted, washed twice, and resuspended in PBS to an OD_600_ of 0.5. The GFP fluorescence intensity at 507 nm was measured with a spectrofluorophotometer (PerkinElmer LS-55) using an excitation light at 488 nm.

### Bioinformatics

DinJ-YafQ homologs that exhibit sequence conservation in promoter regions were identified by BlastX search using MK544944.1 as query. The nucleotide sequences of the regions upstream of the corresponding genes were aligned using ClustalO at default settings at the EMBL-EBI webportal (https://www.ebi.ac.uk/Tools/msa/clustalo/; Madeira et al. 2019) and improved by manual editing. Sequence alignment was rendered with the web tool ESPript 3.0 (https://espript.ibcp.fr; Robert and Gouet 2014).

DinJ and YafQ model structures were obtained by homology modeling using SWISS-MODEL (https://swissmodel.expasy.org; Bordoli et al. 2009). *E. Coli* DinJ (PDB ID: 4Q2U; Ruangprasert et al. 2014) and *E. Coli* YafQ (PDB ID: 4ML2; Liang et al. 2014) were used as templates. To create a heterotetrameric YafQ-(DinJ)2-YafQ complex, DinJ and YafQ models were aligned with the crystal structure of the *E. coli* DinJ-YafQ complex (PDB ID: 4Q2U). A double helical secondary structure of DNA harboring the sequence (TTATCCCACTGTGTTTACATTGGGATAA) of *dinJ-yafQ* promoter was generated with the SCIFBio tool (http://www.scfbio-iitd.res.in/software/drugdesign/bdna.jsp; Arnott et al. 1976). To construct a structural model for the binding of two YafQ-(DinJ)2-YafQ complexes to the promoter inverted repeat, the crystal structure of Arc-DNA complex (PDB ID: 1PAR; Raumann et al. 1994) was used as a template. Because the centers of the two inverted sequences of *dinJ-yafQ* promoter are separated by 16 bp, corresponding to one and a half turn of the DNA helix, the two hemisites are accessible on the opposite sides of the DNA helix. Therefore, one DinJ-YafQ complex was aligned with one Arc dimer in the center of one hemisite, while the other one was manually positioned in the center of the other hemisite at the opposite side of the DNA helix. Alignment and manual refinement of structural models were performed with PyMOL (The PyMOL Molecular Graphics System, Version 1.3 Schrödinger, LLC.).

## Results

### Oligomeric state of purified DinJ, YafQ, and DinJ-YafQ complex

**Fig. 1:**
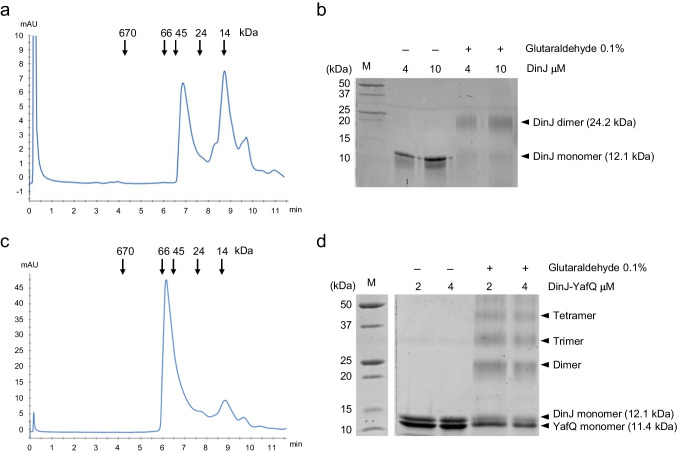
Oligomeric state characterization of purified DinJ and DinJ-YafQ complex. **a** Size-exclusion chromatography (Superdex 200 Increase 5/150 GL column) elution profile of purified DinJ. The two major peaks (elution time: 6.87 and 8.77 min) correspond to an apparent MW of 35 and 14.6 kDa, respectively. MW and retention time of the protein markers are as follows: thyroglobulin (670 kDa; 4.27 min), BSA (66 kDa; 6.13 min), ovalbumin (45 kDa; 6.55 min), trypsinogen (24 kDa; 7.72 min), lysozyme (14.6 kDa; 8.79 min). **b** SDS-PAGE analysis of DinJ (4 and 10 µM) before and after cross-linking with glutaraldehyde 0.1%. Arrowheads indicate the oligomeric states of the cross-linked species as expected from the predicted molecular mass. M, protein marker. **c** Size-exclusion chromatography (Superdex 200 Increase 5/150 GL column) elution profile of purified DinJ-YafQ. The major peak (elution time: 6.16 min) corresponds to an apparent MW of 57 kDa. MW and retention time of the protein markers are as in **a**.** d** SDS-PAGE analysis of DinJ-YafQ (2 and 4 µM) before and after cross-linking with glutaraldehyde 0.1%. Arrowheads indicate the oligomeric states of the different cross-linked species as expected from the predicted molecular mass. M, protein marker

Recombinant DinJ, YafQ, and DinJ-YafQ complex from *L. paracasei* 4366 and 2333 were purified as described in “Materials and methods.” To determine the oligomeric form of the proteins in solution, His-tagged DinJ (12.1 kDa), His-tagged YafQ (13.6 kDa), and His-tagged DinJ-YafQ (12.1–11.4 kDa) were subjected to analytical size-exclusion chromatography. The elution profile of DinJ showed two different peaks, corresponding approximately to 14 and 35 kDa that likely represent the monomeric and oligomeric forms of DinJ (Fig. 1a). Cross-linking with glutaraldehyde shows the presence of two bands corresponding to DinJ monomer and dimer, with the latter being the predominant form (Fig. 1b). Thus, in size-exclusion chromatography DinJ dimers migrate with an apparent molecular mass of 35 kDa presumably due to the elongated structure of the protein. The elution profiles of YafQ showed one major peak corresponding to ~20 kDa (Fig. S2), while the elution profile of the DinJ-YafQ complex showed one major peak corresponding to ~57 kDa (Fig. 1c). These data suggest that YafQ is likely a monomer, while DinJ-YafQ complex forms hetero-oligomers. Consistently, DinJ-YafQ cross-linking experiments showed multiple bands on SDS-PAGE corresponding to oligomers comprising dimers, trimers, and tetramers (Fig. 1d). These results are in accordance with the previously reported oligomeric state characterization of DinJ-YafQ from *E. coli* (Liang et al. 2014; Ruangprasert et al. 2014).

### Ribonuclease activity of purified YafQ

**Fig. 2:**
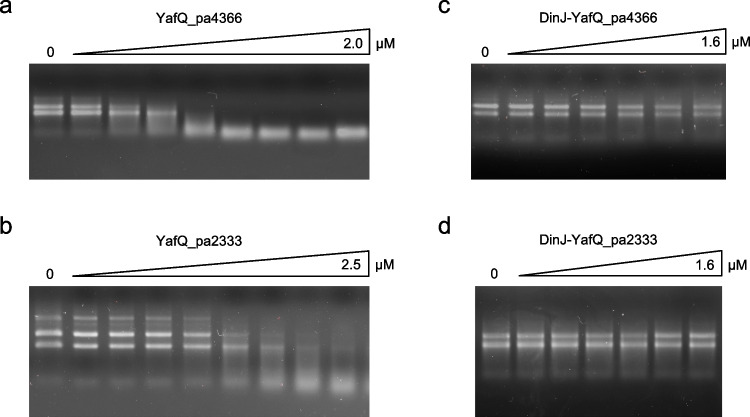
YafQ RNase activity and inhibition by DinJ. Agarose gel electrophoresis showing ribosomal RNA (16S and 23S) cleavage assays at increasing protein concentrations. **a** YafQ_pa4366 (0, 14, 68, 135, 270, 540, 800 nM, and 1.35, 2 µM). **b** YafQ_pa2333 (0, 17, 85, 170, 284, 680 nM, and 1, 1.7, 2.5 µM). **c** DinJ-YafQ_pa4366 (0, 27, 135, 270, 540 nM, and 1.1, 1.6 µM). **d** DinJ-YafQ_pa2333 (0, 27, 135, 270, 540 nM, and 1.1, 1.6 µM)

The ribonuclease activity of YafQ_pa4366 and YafQ_pa2333 was investigated by evaluating the degradation of total RNA isolated from the corresponding strains as described in Levante et al. (2019). After 15 min incubation at RT with an increasing amount of toxin, samples were analyzed by agarose gel electrophoresis. As shown in Fig. 2, both YafQ_pa4366 and YafQ_pa2333 display a clear ribonuclease activity (Fig. 2a, b) which is strongly inhibited by the presence of the antitoxin DinJ (Fig. 2c, d). In particular, the 16S and 23S bacterial rRNAs are completely degraded in the presence of 270 nM YafQ_pa4366 (Fig. 2a), while complete degradation is not observed even in the presence of 2.5 μM YafQ_pa2333 (Fig. 2b). This result demonstrates that the active site D72G substitution reduces RNase activity and corroborates previous growth assays of *E. coli* expressing either YafQ_pa4366 or YafQ_pa2333 (Ferrari et al. 2019).

### DinJ and DinJ-YafQ bind to their operon promoter region

**Fig. 3:**
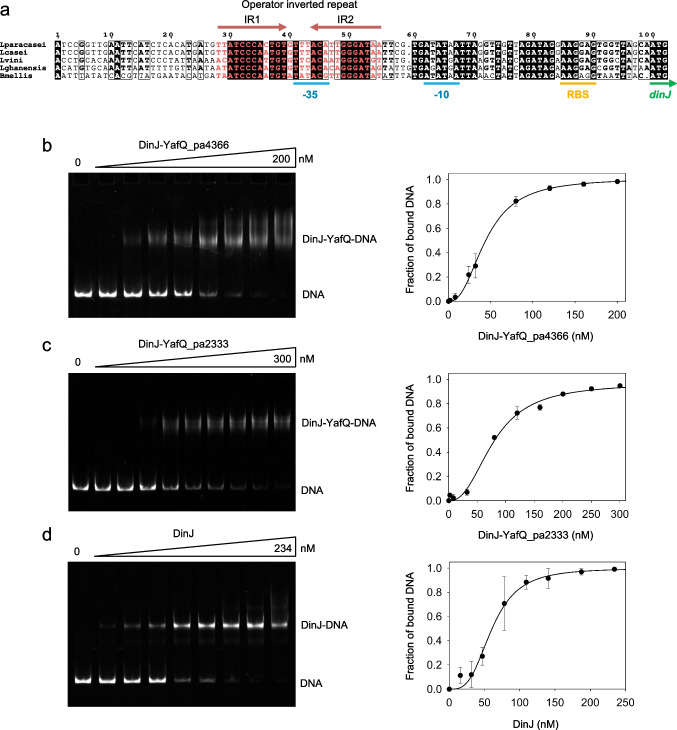
DNA-binding activity of DinJ-YafQ complex and DinJ antitoxin. **a** Multiple alignment of the *dinJ-yafQ* promoter region in different lactic acid bacteria (*Lacticaseibacillus paracasei —* NZ_ANKJ01000027.1:20187-20337; *Lacticaseibacillus casei —* NZ_AZOE01000004.1:33888-34038; *Liquorilactobacillus vini —* NZ_AYYX01000178.1:125-275; *Liquorilactobacillus ghanensis —* NZ_AZGB01000015.1:869-1019; *Bombilactobacillus mellis —* NZ_JAAEEA010000002.1:338150-338300). The inverted repeat sequences are in red with the two hemisites (IR1 and IR2) marked by arrows; the −35 and −10 promoter elements, the ribosome-binding site (RBS) and the *dinJ* starting codon are marked by blue, orange, and green lines, respectively. **b–d** Polyacrylamide gel electrophoresis (left panel) and densitometric analysis (right panel) showing the band-shift of a 194-bp fluorescently labeled DNA fragment harboring *dinJ-yafQ* promoter with **b** DinJ-YafQ_pa4366 (protein concentration: 0, 2, 8, 24, 32, 80, 120, 160, 200 nM), **c** DinJ-YafQ_pa2333 (protein concentration: 0, 2, 8, 32, 80, 120, 160, 200, 250, 300 nM), and **d** DinJ_pa4366 (protein concentration: 0, 16, 32, 48, 78, 109, 140, 187, 234 nM). Data points represent the mean of three independent experiments ± *SD*. Data are fitted with the Hill equation reported in “Materials and methods”

Several studies have reported that type II antitoxins and TA complexes act as transcriptional regulators of the TA operon (Fraikin et al. 2020). In particular, in *E. coli*, DinJ and DinJ-YafQ bind to a 20-bp inverted repeat sequence overlapping the −10 promoter element of *dinJ-yafQ* locus, leading to transcription repression of the operon (Ruangprasert et al. 2014). Similarly, analysis of the region upstream the *dinj-yafQ* locus of *L. paracasei* 4366, highlighted an inverted repeat sequence of 28 nucleotides overlapping the −35 element of the identified *dinJ-yafQ* promoter (Levante et al. 2019). The inverted repeat is highly conserved among other lactic acid bacteria and is formed by two hemisites of 12 bp separated by a 4-bp spacer (Fig. 3a).

To address a possible regulatory role of the identified inverted repeat, we have employed gel mobility shift assays (EMSA) to investigate the binding of DinJ and DinJ-YafQ complex to the promoter region. A 194-bp long fluorescently labeled DNA fragment, with the inverted repeat positioned in the center, was incubated with either DinJ or DinJ-YafQ complex and analyzed by a 6% native polyacrylamide gel electrophoresis. As shown in Fig. 3b, DinJ-YafQ_pa4366 shifts the DNA fragment in a concentration-dependent manner from 0 to 200 nM. At the higher protein concentrations, a moderate degree of super shift was also observed. Fluorescence intensity quantification of the free DNA band resulted in a sigmoidal binding isotherm best fitted with a Hill equation. An apparent *K*_Dapp_ of approximately 44 ± 7 nM and a Hill constant of 2.4 ± 0.2 were determined from the fitting. Similarly, binding of DinJ-YafQ_pa2333 to DNA resulted in a *K*_Dapp_ of 79 ± 3 nM and a Hill constant of 2.4 ± 0.5 (Fig. 3c). Gel retardation assay was also performed to analyze the binding of purified DinJ to DNA. As shown in Fig. 3d, DinJ binds the promoter DNA cooperatively and with a *K*_Dapp_ of 61 ± 11 nM and a Hill constant of 3.7 ± 0.2. These results indicate that both DinJ and DinJ-YafQ complex bind the dsDNA target with high affinity and in a cooperative manner. It should be noted that all our EMSA assays were performed with N-terminal His-tagged DinJ, suggesting that the presence of the extra amino terminal tag does not prevent binding of DinJ or DinJ-YafQ to the DNA. This observation is at variance with previous data obtained in *E. coli*, where DNA binding was inhibited by the amino terminal His-tag but not by the carbossi-terminal His-tag (Armalytė et al. 2012).

### DinJ-YafQ binds the promoter DNA with different stoichiometries

**Fig. 4:**
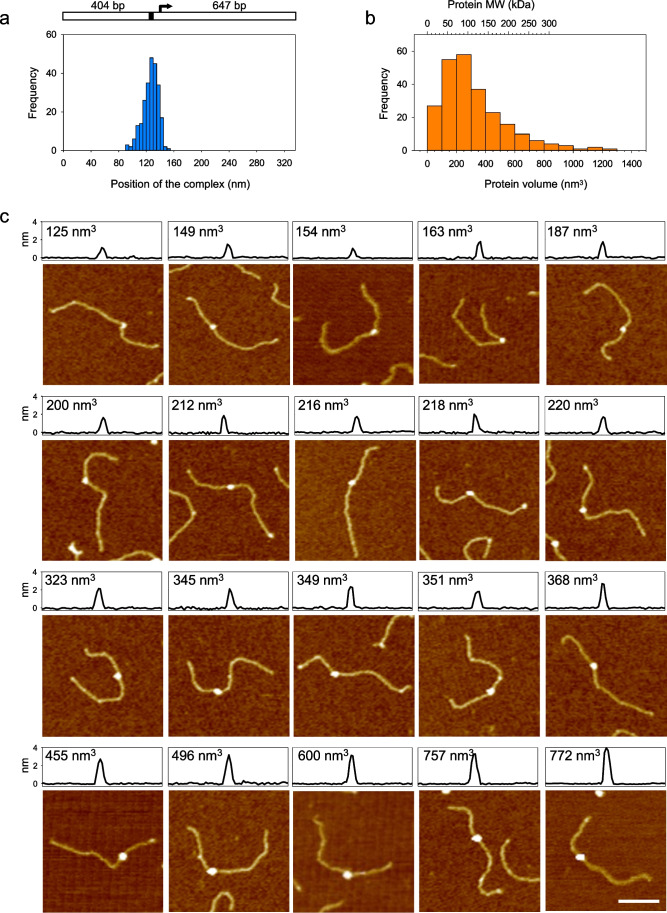
AFM analysis of DinJ-YafQ/DNA complexes. **a** Distribution of DinJ-YafQ binding position along the 1051-bp DNA template depicted above the graph. The operator inverted repeat is shown as a black mark while the promoter +1 is represented by an arrow. 404 bp and 647 bp represent the distance from the center of the inverted repeat to either DNA ends. **b** Distribution of volumes of specific DinJ-YafQ complexes. The scale in kDa at the top of the graph represents the calibration curve shown in Fig. S1b. **c** Montage of specific DinJ-YafQ complexes ordered by volume. The image profile of each complex is shown at the top of the panel. Bar size, 100 nm

To investigate the interaction between DinJ-YafQ and the TA promoter, we have employed atomic force microscopy (AFM) imaging. DinJ-YafQ/DNA complexes were assembled in solution, cross-linked with glutaraldehyde, and deposited onto freshly-cleaved mica as described in “Material and methods.” The glutaraldehyde cross-linking was necessary to prevent complex dissociation upon adhesion to the hydrophilic mica surface. A linear DNA fragment of 1051 bp containing the TA promoter with the operator site positioned at 404 bp from one end was used as a template. As shown in Fig. S3, several DNA fragments have a nucleoprotein complex at the expected position along the DNA template. By measuring the distance of the complex from the DNA ends, we could confirm that DinJ-YafQ preferentially binds DNA in a position corresponding to the operator inverted repeat (Fig. 4a). From a visual inspection of the images, we also noticed that the nucleoprotein complexes formed at the operator site were different in size. Some of them were very small, some were of medium size, and some were quite large. The DinJ-YafQ dimer has a molecular mass of 23.5 kDa which is at the lower limit of AFM detection capability; thus, the complexes depicted in Fig. S3 should be formed by DinJ-YafQ oligomers. As previously reported, the stoichiometry of a nucleoprotein complex can be obtained by measuring the volume of the protein/DNA complexes imaged by AFM. The molecular mass of the complexes is then inferred by means of a calibration curve obtained with proteins of known molecular weight (Fig. S1). Thus, we measured the volume of many specific complexes using the procedure detailed in “Materials and methods” and the data are plotted in Fig. 4b. The distribution of volumes is wide, ranging from 20 up to 1200 nm^3^, with a peak around 250 nm^3^. Based on the calibration curve (Fig. S1), these volumes correspond to a molecular mass ranging from 50 to more than 300 kDa (the upper limit of our calibration curve). This distribution indicates that DinJ-YafQ binds the operator site to repress transcription of its own promoter by forming oligomers with a different stoichiometry. Most of the complexes have a molecular mass that is compatible with the formation of nucleoprotein complexes formed by two, three, or four DinJ-YafQ dimers. Very small or very large complexes were less frequently observed. Figure 4c depicts a gallery of DinJ-YafQ/DNA complexes ordered by volume in which the first row should represent complexes with two DinJ-YafQ dimers, the second row complexes with three DinJ-YafQ dimers, the third row complexes with four DinJ-YafQ dimers, and the fourth row complexes with six or more DinJ-YafQ dimers. These data indicate that DinJ-YafQ binds the operator site by forming oligomers with different stoichiometries and corroborate the evidence for binding cooperativity.

### DinJ-YafQ competes with RNA polymerase for promoter occupancy

**Fig. 5:**
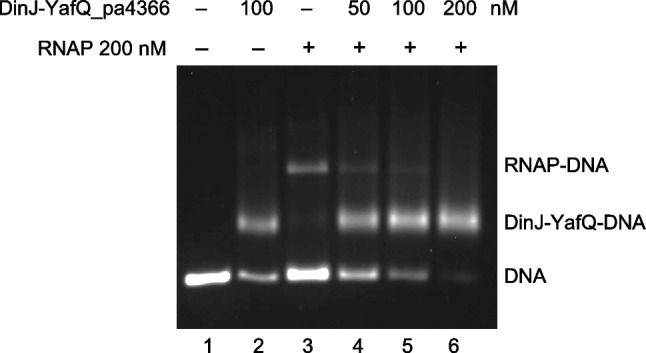
RNA polymerase promoter binding competition by DinJ-YafQ. Agarose gel electrophoresis showing the band-shift of a 194 bp fluorescently labeled DNA fragment harboring the *dinJ-yafQ* promoter (lane 1) with DinJ-YafQ_pa4366 (lane 2), with RNAP in the absence (lane 3) or in the presence of increasing concentrations of DinJ-YafQ (lanes 4, 5, and 6)

To validate the hypothesis that DinJ-YafQ binds its own promoter and represses transcription, we performed RNAP competition binding assays using EMSA. Due to the unavailability of purified *Lactobacillus* RNAP, commercially available *E. coli* sigma-70 RNAP holoenzyme was used. The −10 and −35 elements of the *dinJ-yafQ* promoter have a sequence nearly identical to the consensus, with a spacer of 16 bp which is also found in many *E. coli* promoters. In addition, as shown below, in vivo transcription demonstrates that the *L. paracasei dinJ-yafQ* promoter is actively transcribed in the C41 (DE3) pLysS *E. coli* strain. As shown in Fig. 5, under conditions that favor open promoter complex formation, binding of RNAP to fluorescently-labeled DNA determines a pronounced shift in gel migration due to the larger size of RNAP compared to DinJ-YafQ. Incubation of the DNA with DinJ-YafQ prior to RNAP addition caused the disappearance of the band corresponding to the RNAP promoter complex in a DinJ-YafQ concentration-dependent manner. This result is consistent with the hypothesis that binding of DinJ-YafQ to the promoter region represses transcription by making the promoter inaccessible to RNAP. In a similar competition experiment, binding of RNAP to the TA promoter was also inhibited by DinJ alone in a concentration-dependent manner (Fig. S4). In this case, shifted bands appear smeared because the DinJ-DNA complex partially dissociates when incubated at 37 °C, a step that favors the formation of the RNAP open promoter complex (Fig. S4a). In a control experiment, DinJ-YafQ was unable to compete with the binding of RNAP to a DNA fragment harboring a lambda PR promoter (Fig. S4b), thus confirming that the competition is caused by an interaction with the DNA rather than with the RNAP.

### A conserved arginine in the RHH DNA-binding motif of DinJ is essential for DNA binding

**Fig. 6:**
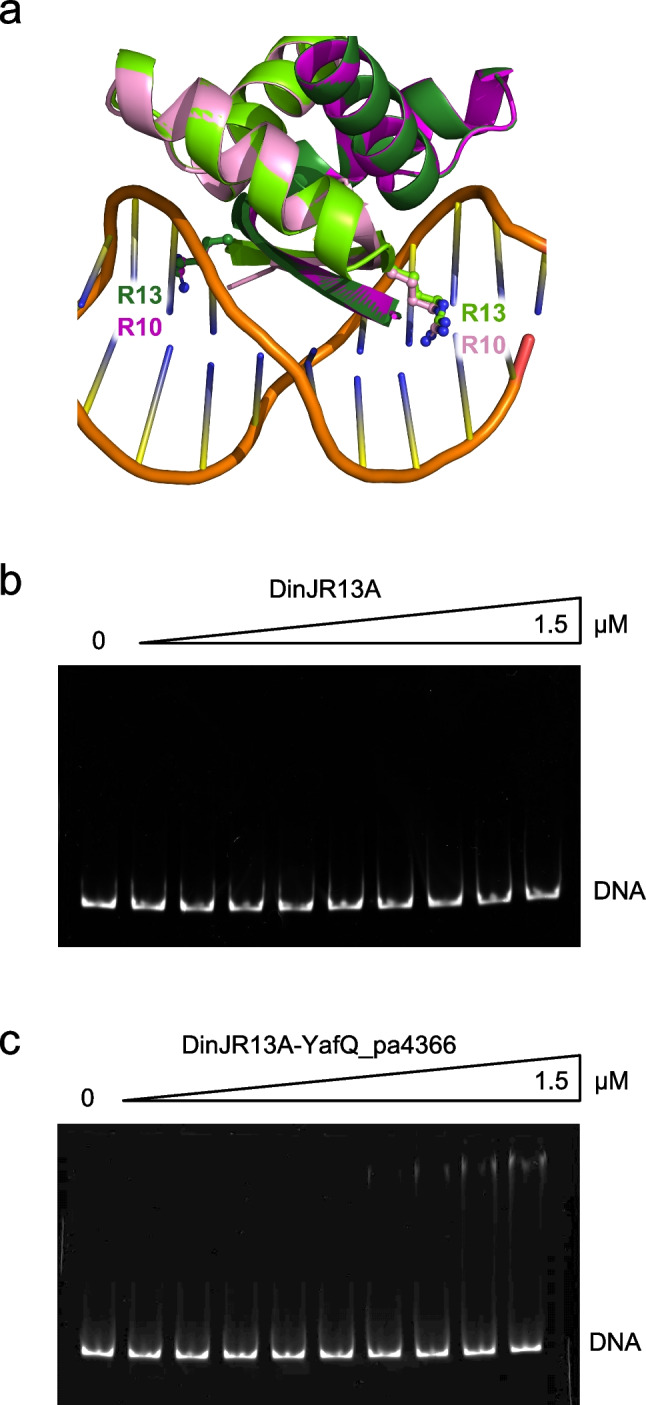
Effect of the DinJ R13A substitution on DNA binding. **a** Modeling of the DinJ DNA binding domain based on the structure of DNA-Arc repressor complex (PDB id: 1PAR) obtained as described in “Materials and methods.” Superimposition of the dimeric *L. paracasei* DinJ model structure (chain A, dark green; chain B, light green) with dimeric *E. coli* DinJ structure (chain A, magenta; chain B, pink; PDB id: 4Q2U). Arginines of the RHH DNA-binding motif are shown in sticks and labeled. DNA backbone is shown in orange. Polyacrylamide gel electrophoresis showing absence of binding to the TA promoter region of DinJR13A (**b**) and DinJR13A-YafQ_pa4366 (**c**). In both gels, protein concentrations are as follows: 0, 50, 100, 200, 400, 600, 800, 1000, 1200, 1500 nM

From the sequence and structure alignment of *E. coli* and *L. paracasei* DinJ, we noticed that the arginine residue R13, located in the RHH DNA-binding motif of *L. paracasei* DinJ, overlaps with R10 of *E. coli* DinJ (Fig. 6a) that has been found to participate in the interaction with the inverted repeat of the TA promoter (Ruangprasert et al. 2014). Based on this structural evidence, we have substituted DinJ R13 with alanine and analyzed the effect of this mutation on DNA binding. First, we verified that DinJ R13A can still form a stable inactive complex with YafQ by RNase cleavage assay (Fig. S5). In addition, as shown in Fig. 6b, the R13A substitution resulted in a complete loss of DNA binding by DinJ up to a protein concentration of 1.5 µM. A similar result is obtained with DinJR13A-YafQ_pa4366 complex, even though in this case, we observed the appearance of a retarded band at the higher protein concentrations (Fig. 6c). These results highlight the essential role of this arginine residue in the recognition of the operator site also for *L. paracasei* DinJ. Such a strong effect on the binding affinity by a single amino acid substitution may also reflect the cooperative binding of the protein to the cognate site.

### DinJ and DinJ-YafQ regulate the TA operon promoter in vivo

**Fig. 7:**
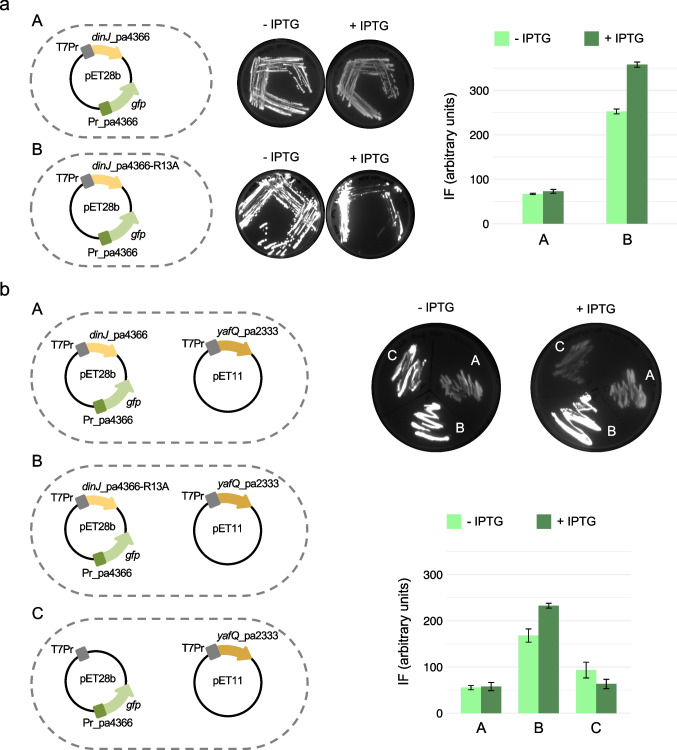
In vivo transcriptional repression by DinJ and DinJ-YafQ. **a** Recombinant plasmids used to transform C41 (DE3) pLysS *E. coli* cells to analyze the regulatory effect of DinJ (A) or DinJR13A (B) on the *dinJ-yafQ* promoter. The GFP fluorescence was analyzed on solid (LB-agar plates) and in liquid (LB medium; bar plot) in the absence and in the presence of 0.5 mM IPTG. **b** Recombinant plasmids used to co-transform C41 (DE3) pLysS *E. coli* cells to analyze the regulatory effect of DinJ-YafQ (A) or DinJR13A-YafQ (B) or YafQ (C) on the *dinJ-yafQ* promoter. The GFP fluorescence was analyzed on solid (LB-agar plates) and in liquid (LB medium; bar plot) in the absence and in the presence of 0.5 mM IPTG. T7Pr, T7 promoter; Pr_pa4366, *dinJ-yafQ* promoter region of *L. paracasei* 4366; *dinJ*_pa4366 and *dinJ*_pa4366-R13A, ORF of *L. paracasei* 4366 *dinJ* and R13A *dinJ* mutant, respectively; *yafQ*_pa2333, ORF of *L. paracasei* 2333 *yafQ*; *gfp*, ORF of reporter *gfp* gene. The fluorescence intensity (IF) represents the mean of three independent experiments ± *SD*. For the sake of clarity, bacterial cells are outlined by a dashed line

Next, we performed a set of in vivo experiments aimed to validate the hypothesized repression of the *dinJ-yafQ* promoter by DinJ-YafQ, using GFP as reporter gene. GFP has several advantages such as its fluorescence; its stability; it requires no substrate, cofactor, or additional proteins for detection; and it has been successfully used to monitor the regulation of the *dinJ-yafQ* promoter in *Tetragenococcus halophilus* (Luo et al. 2021). Thus, we constructed several recombinant plasmids using pET28b and pET11b vectors and analyzed the fluorescence intensity of transformed or double-transformed C41 (DE3) pLysS *E. coli* strains. Experiments aimed to analyze DinJ activity are reported in Fig. 7a. The construct pET28b containing *dinJ*_pa4366 sequence under the control of the T7 promoter (T7Pr) and the *gfp* ORF fused to the *dinJ-yafQ* promoter (Pr_pa4366-*gfp*) was used to transform *E. coli* cells. Growth assays in the absence and in the presence of IPTG were carried out on solid and in liquid medium. As shown in Fig. 7a-A, on solid medium, GFP fluorescence decreases in the presence of 0.5 mM IPTG, suggesting a downregulation of the *dinJ-yafQ* promoter. This reduction of fluorescence intensity is not observed in liquid medium (histogram in Fig. 7a). A similar construct in which *dinJ*_pa4366 has been replaced with *dinJ*_pa4366 R13A mutant resulted in a much higher fluorescence intensity both on solid and in liquid medium as expected by the compromised DNA binding affinity of this DinJ mutant. This result further suggests that even in the absence of IPTG, DinJ basal expression is sufficient to repress transcription of its own promoter. To investigate the role of YafQ in the regulation of the *dinJ-yafQ* promoter, the *E. coli* strains described above were co-transformed with a pET11b vector containing the YafQ_pa2333 coding sequence under the control of T7Pr (Fig. 7b-A, B). For these experiments, we used YafQ from the *L. paracasei* strain 2333 because it is less toxic compared to YafQ_pa4366 due to the D72G substitution, while DinJ has the same sequence in both strains. In the case of *wt* DinJ, co-expression of YafQ_pa2333 has little or no effect on the fluorescence intensity both on solid and in liquid medium, either with or without IPTG induction. A similar result was obtained in the case of DinJ R13A co-expressed with YafQ_pa2333, even though the fluorescence intensity of the in liquid culture is higher (histogram in Fig. 7b). As a control experiment, we analyzed the fluorescence intensity of an *E. coli* strain co-transformed with pET28b carrying Pr_pa4366-*gfp* and pET11b vector containing the YafQ_pa2333 coding sequence under the control of T7Pr (Fig. 7b-C). In the absence of IPTG, the fluorescence intensity is higher with respect to that of the strain expressing the *wt* complex but lower than that of the strain expressing DinJR13A-YafQ_pa2333 complex. In the presence of IPTG, the fluorescence intensity decreases both on solid and in liquid medium, an effect that may be attributed to the RNase activity of YafQ. Further control experiments with an *E. coli* strain carrying only the pET28b with Pr_pa4366-*gfp* resulted in a low percentage of fluorescent cells (Fig. S6), revealing a possible toxic effect of GFP when expressed at high concentrations (Kain 2005). This observation indicates that the *dinJ-yafQ* promoter is highly transcribed in the *E. coli* host.

The unexpected high expression-related toxicity of GFP, that adds up to the intrinsic YafQ toxicity, complicates the interpretation of the *in vivo* experimental results. However, these data demonstrate that both *wt* DinJ and *wt* DinJ-YafQ complex repress transcription of the TA promoter to a similar extent. The higher fluorescence intensity observed with DinJ R13A with respect to *wt* DinJ can be explained with a weaker repression of the TA promoter due to a lower DNA binding affinity. The fluorescence of DinJ R13A is also higher than that of *E. coli* transformed with pET28b harboring only Pr_pa4366-*gfp* (Fig. S6); this can be explained by a weak repression of the TA promoter by DinJ R13A that may be sufficient to reduce GFP concentration to non-toxic levels. In the case of DinJ R13A, the presence of YafQ increases promoter repression suggesting a stronger DNA binding of the complex compared to DinJ alone.

## Discussion

Type II TA systems, such as DinJ-YafQ, are widely distributed among different bacterial species and represent stress-response mechanisms acting by blocking or slowing down essential cell processes to overcome adverse growth conditions. Although TA systems are extensively studied in pathogenic bacteria, little has been reported for lactic acid bacteria in spite of their extensive application in food, nutraceutical, and pharmaceutical industries.

In this study, we investigated the functional role of recombinant DinJ and YafQ from strains 4366 and 2333 of *L. paracasei* isolated from food matrices (Ferrari et al. 2019). Although a purification procedure to obtain the toxin YafQ has been previously reported (Maggi et al. 2021), herein we have optimized several steps of the procedure in order to have higher protein yield and to reduce the purification time. In particular, the substitution of the Talon metal affinity resin with an HisTrap column functionalized with Ni, the use of an automatized chromatography system, and the introduction of a shock refolding step represent the main advantages.

*L. paracasei* 4366 and 2333 YafQ toxicity and RNase activity have been previously studied in vivo in the *E. coli* host (Ferrari et al. 2019). Upon induction of YafQ_pa4366, a significant inhibition of the cell growth and a decrease of the Thioflavin T fluorescence were observed, suggesting an active RNA degradation. On the other hand, induction of YafQ_pa2333 resulted in a negligible growth inhibition and undetectable RNA degradation. This raised the question about the effective role of this TA system in which a YafQ toxin carrying a single D72G substitution in the active site co-exists with a fully conserved DinJ antitoxin, a feature shared with other *L. paracasei* strains (Ferrari et al. 2019). The in vitro RNase activity of YafQ_pa4366 and YafQ_pa2333, reported in the present study, clearly shows that, at difference with the in vivo results, YafQ_pa2333 cleaves ribosomal RNA albeit with reduced enzymatic activity compared to YafQ_pa4366. These experiments also show that the RNase activity of YafQ from both strains is completely neutralized by DinJ. These results demonstrate that *L. paracasei* 2333 DinJ-YafQ forms an active TA system but with a reduced toxicity.

As reported for other type II TA systems in general, and for *E. coli* DinJ-YafQ in particular, either the antitoxin alone or the toxin-antitoxin complex binds an operator site within the TA operon promoter to repress transcription initiation (Liang et al. 2014; Ruangprasert et al. 2014; Fraikin et al. 2020). The putative DinJ-YafQ operator site, predicted in *L. paracasei* strains by sequence conservation analysis and structural similarity with the *E. coli* operator (Levante et al. 2019), contains an inverted repeat formed by two hemisites of 12 bp separated by a 4-bp spacer, whereas the *E. coli* operator inverted repeat is formed by two hemisites of 9 bp separated by a 2-bp spacer (Ruangprasert et al. 2014). The former operator overlaps the −35 promoter element while the latter overlaps the −10 promoter element.

**Fig. 8:**
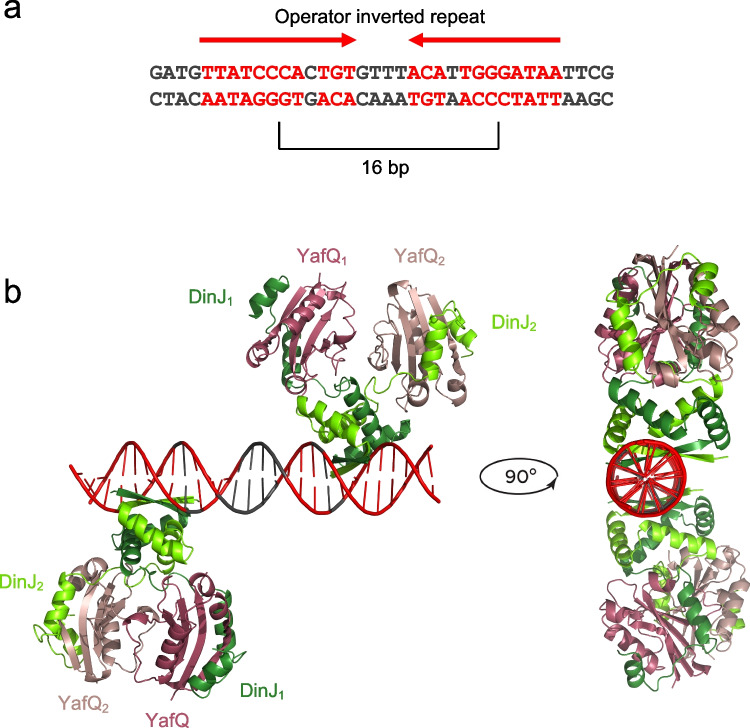
Model of DinJ-YafQ binding to DNA inverted repeat. **a** Sequence of *L. paracasei* inverted repeat (red). **b** Structural model for the binding of a heterotetrameric DinJ-YafQ complex to IR1 and IR2 hemisites obtained as described in “Material and methods”

Our DNA binding assays show that the DinJ-YafQ complex of both 4366 and 2333 strains binds the operator site cooperatively and with an affinity in the nanomolar range. Cooperative DNA binding has been previously suggested for the RelBE system (Bøggild et al. 2012), but it has been ruled out for the *E. coli* DinJ-YafQ system because of the steric hindrance between the two DinJ-YafQ tetramers bound to each hemisite disclosed by a structural model (Ruangprasert et al. 2014). However, the larger size of the inverted repeat and the larger intervening spacer observed in the *L. paracasei* operator should allow the simultaneous accommodation of two DinJ-YafQ tetramers. By using the same approach followed in Ruangprasert et al. (2014), we have built a structural model to examine the relationship between two DinJ-YafQ tetramers bound to each hemisite of the *L. paracasei* operator (Fig. 8). In this case, the 16 bp distance between the centers of the two inverted sequences moves the major groove binding sites far apart and on the opposite sides of the DNA helix. With this arrangement, binding of two tetramers does not show steric hindrance, thus supporting the formation of DinJ-YafQ oligomers that may account for binding cooperativity. Interestingly, DinJ-YafQ cross-linking shows the formation of different oligomeric forms in solution (Fig. 1), while AFM images reveal that DinJ-YafQ forms nucleoprotein complexes with different molecular mass (i.e., stoichiometry) at the position of the operator site (Fig. 4). AFM data suggest that the most frequent complexes observed are compatible with the molecular mass of two, three, or four DinJ-YafQ complexes; however, larger oligomeric states are also present.

As shown for other DinJ-YafQ TA systems (Liang et al. 2014; Luo et al. 2021), DNA binding is driven by DinJ and, most probably, by the RHH motif as proposed in the structural model reported in (Ruangprasert et al. 2014) and in Fig. 8. Our data confirm that DinJ alone binds the operator site cooperatively with an affinity in the nanomolar range, similarly to that observed for the DinJ-YafQ complex. Conversely, YafQ alone does not bind the operator site (data not shown). Attempts to image DinJ-DNA complexes by AFM were unsuccessful probably because of the lower protein stability and the small DinJ molecular mass (12.1 kDa) that is below the detection limit of our AFM microscope.

In support of the proposed RHH motif involvement in the DNA binding of DinJ is the observation that the R13A mutation of *L. paracasei* DinJ, structurally equivalent to the R10A mutation of *E. coli* DinJ (Ruangprasert et al. 2014), completely abolishes DNA binding at all protein concentrations tested and with DinJ either alone or in complex with YafQ (Fig. 6).

Last, we present in vitro and in vivo experiments aimed to evaluate *dinj-yafQ* promoter repression by DinJ and DinJ-YafQ. Given the overlap between the operator site and the −35 promoter element, we reasoned that the strong DNA binding shown by DinJ-YafQ might be sufficient to inhibit RNAP binding to the promoter and that this inhibition might be detectable in an EMSA assay. Indeed, the formation of *E. coli* RNAP-promoter complexes is inhibited when the promoter DNA is preincubated with either DinJ-YafQ or DinJ, suggesting that transcriptional repression exerted by DinJ-YafQ occurs by hindering RNAP closed promoter complex formation.

Interpretation of experiments aimed to validate transcriptional repression by DinJ-YafQ in vivo was problematic due to an unexpected toxicity of the reporter GFP when expressed at high concentrations. In a previous study, a construct with a *gfp* ORF under the control of a *Tetragenococcus halophilus dinJ-yafQ* promoter proved to be a versatile and useful recombinant system to monitor promoter regulation by DinJ and DinJ-YafQ (Luo et al. 2021). However, *E. coli* cultures grown both on solid and in liquid medium show that DinJ and DinJ-YafQ act as repressors by reducing GFP expression. In fact, the growth of *E. coli* cells right after transformation with a plasmid harboring only the *gfp* ORF under the control of the *dinJ-yafQ* promoter (Pr_pa4366-*gfp*) resulted in a small number of colonies, most of which were not fluorescent. Attempts to transfer these colonies onto fresh plates resulted in the absence of growth (data not shown). These findings indicate that the *gfp* reporter gene is highly expressed in the *E. coli* host, thus suggesting that Pr_pa4366 is a strong and efficiently transcribed promoter. Transformation with a plasmid harboring Pr_pa4366-*gfp* and *dinJ* under the control of the inducible T7 promoter resulted in an increased number of colonies all of which were fluorescent. The small or negligible fluorescence difference between induced and uninduced cells can be justified by the basal transcription of the T7 promoter. Co-transformation of this recombinant *E. coli* strain with an inducible plasmid expressing YafQ gave similar results, thus suggesting a comparable repression activity of DinJ and DinJ-YafQ. Based on these observations, we thought that the expression of the mutant form DinJR13A, either alone or in complex with YafQ, should lead to a “no repression” phenotype. Surprisingly, this hypothesis has been refuted by the observation that these recombinant *E. coli* strains displayed a more pronounced cell growth and a significantly higher fluorescence intensity. This result suggests that, albeit in vitro DNA binding was completely abolished by the R13A substitution, in vivo, a probably higher DinJ-YafQ concentration within the cell may lead to a partial repression of the *dinJ-yafQ* promoter with a consequently lower GFP expression, not harmful for the cell survival.

Overall, the data demonstrate that DinJ-YafQ TA systems from different *L. paracasei* strains have variable levels of toxicity due to a different RNase activity of YafQ orthologs. Both DinJ and DinJ-YafQ complex regulate transcription of their own operon by binding to an inverted repeat spanning over the promoter. At variance with the *E. coli* system, the size and arrangement of the inverted sequences and our DNA binding evidence open the possibility for a cooperative regulation mechanism.

## Supplementary Information

Below is the link to the electronic supplementary material.

Supplementary file1 (PDF 652 KB)

## Data Availability

The datasets generated during and/or analyzed during the current study are available from the corresponding author on reasonable request.
