# Supplementary material for: Functional characterization and transcriptional repression by Lacticaseibacillus paracasei DinJ-YafQ
Source: Appl Microbiol Biotechnol. 2022 Oct 4;106(21):7113–28. doi: 10.1007/s00253-022-12195-4 (PMC9592637; doi:10.1007/s00253-022-12195-4)
Supplement: Supplementary file 1 — Supplementary file1 (PDF 652 KB) [file 253_2022_12195_MOESM1_ESM.pdf]

Applied Microbiology and Biotechnology

Supplementary materials

Functional characterization and transcriptional repression by *Lacticaseibacillus paracasei* DinJ-YafQ

Aleksandra Anna Bonini[1], Stefano Maggi[1], Giulia Mori[1], Dario Carnuccio[1], Danila Delfino[1], Davide Cavazzini[1], Alberto Ferrari[2], Alessia Levante[2], Yoshihiro Yamaguchi[3], Claudio Rivetti[1]* and Claudia Folli[2]*

[1]Department of Chemistry, Life Sciences and Environmental Sustainability, University of Parma, 43124 Parma, Italy; [2]Department of Food and Drug, University of Parma, 43124 Parma, Italy; [3]Graduate School of Science, Osaka City University, Sugimoto, Sumiyoshi-ku, Osaka 558-8585, Japan.

*Corresponding author

Co-corresponding authors: Claudia Folli, Claudio Rivetti

Correspondence should be addressed to: claudia.folli@unipr.it or to claudio.rivetti@unipr.it

a

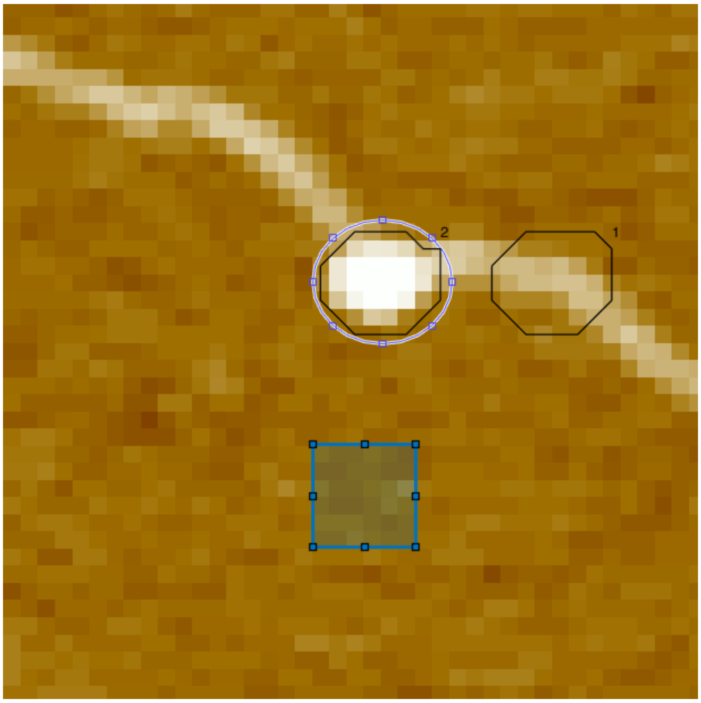

b

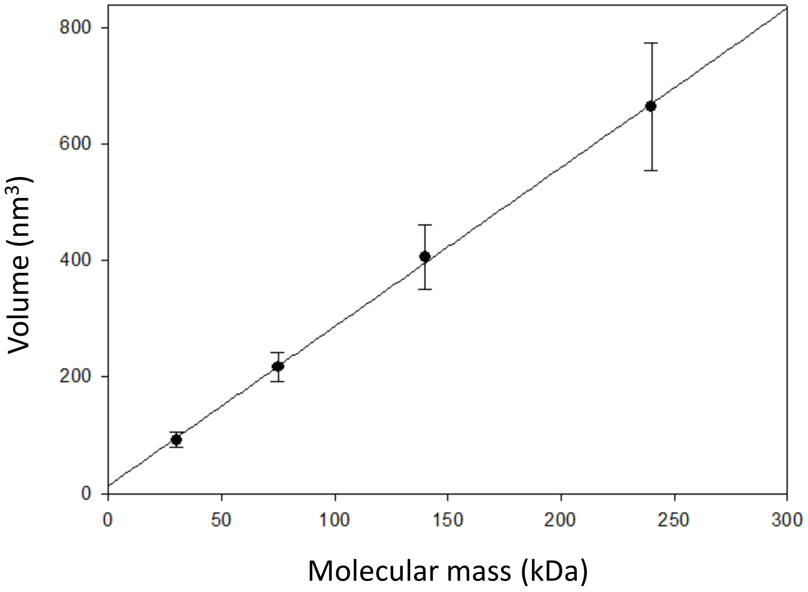

Figure S1 a Schematic representation of the procedure used to measure the volume of DinJ-YafQ complexes. The white ellipse represents the hand-drawn shape used to compute the image mask. The black outlines labeled 1 and 2 delimit the pixels used to measure the volume of the DNA moiety and of the complex, respectively. Pixels within the blue square box are used to compute the reference background. (**b**) Calibration curve used to infer stoichiometry of DinJ-YafQ/DNA complexes. The data points correspond to: Carbonic anhydrase (30 kDa), Conalbumin (75 kDa), Alcohol dehydrogenase (ADH) (140 kDa) and Catalase (240 kDa).

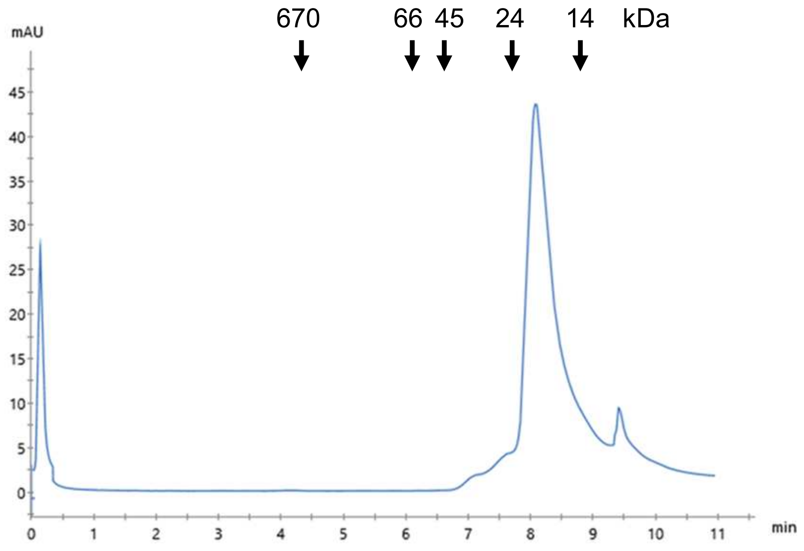

Figure S2 Size-exclusion chromatography (Superdex 200 Increase 5/150 GL column) elution profile of purified YafQ_pa4366. The major peak (elution time: 8.09 min) corresponds to an apparent MW of 20 kDa. MW and retention time of the protein markers are: Thyreoglobulin (670 kDa; 4.27 min), BSA (66 kDa; 6.13 min), Ovalbumin (45 kDa; 6.55 min), Trypsinogen (24 kDa; 7.72 min), Lysozyme (14.6 kDa; 8.79 min).

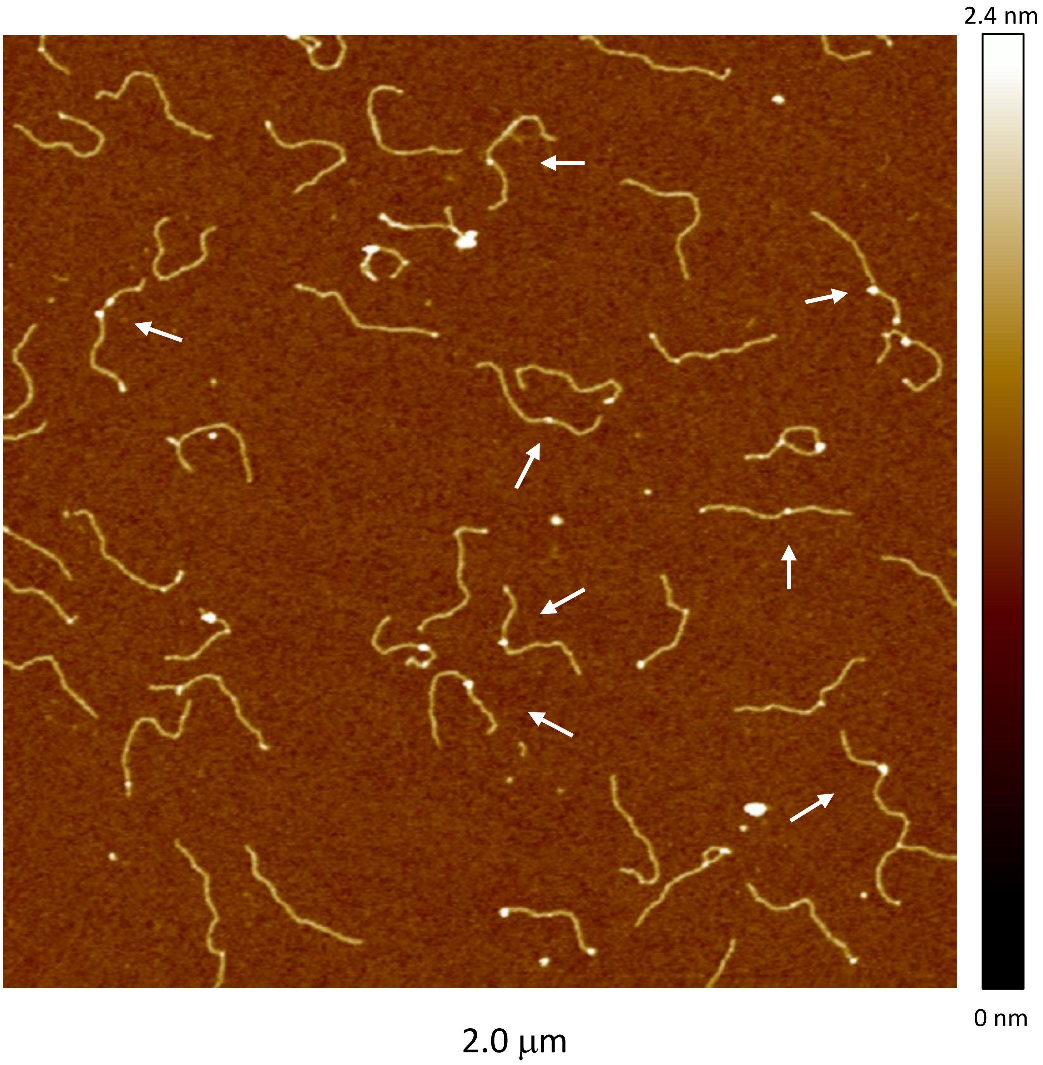

Figure S3. Representative full-scan AFM image of DinJ-YafQ complexes formed with a 1051 bp long DNA fragment. White arrows point to specific complexes.

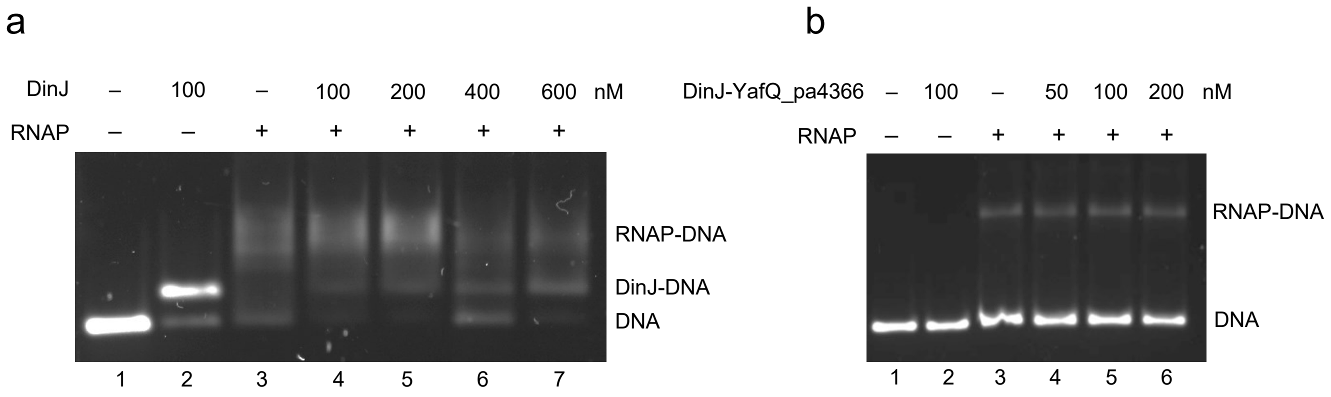

Figure S4 RNA polymerase promoter binding competition. **a** Agarose gel electrophoresis showing the band-shift of a 194 bp fluorescently labeled DNA fragment harboring the dinJ-yafQ promoter (lane 1) with DinJ (lane 2), with RNAP (200 nM) in the absence (lane 3) or in the presence of increasing concentration of DinJ (lanes 4-7). **(b)** Agarose gel electrophoresis showing the absence of competitive binding of DinJ-YafQ_pa4366 and RNAP (200 nM) to a 196 bp fluorescently labeled DNA fragment harboring the λPR promoter.

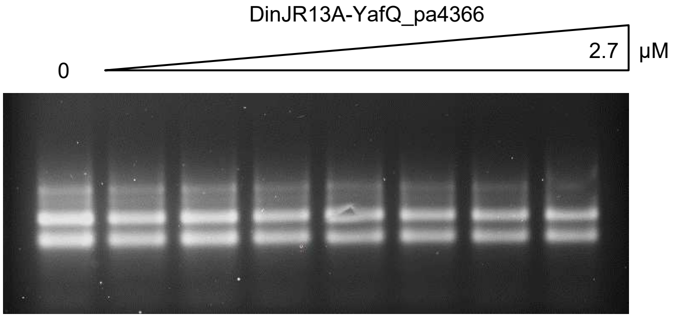

Figure S5 Agarose gel electrophoresis showing absence of RNase activity by DinJR13A-YafQ_pa4366 at increasing protein concentrations (lanes from left to right: 0, 27, 136, 272, 544 nM, and 1.1, 1.6, 2.7 μM).

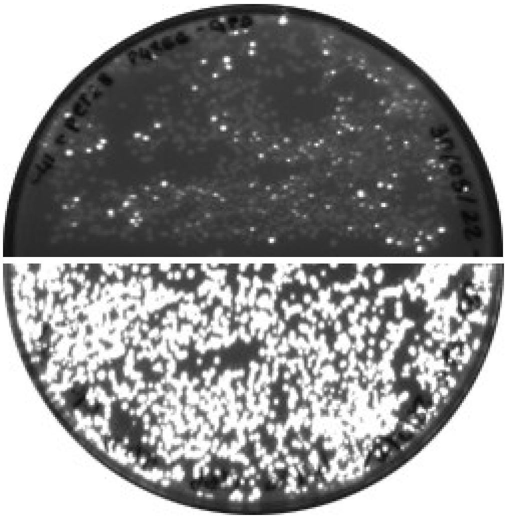

Figure S6 LB agar plates with colonies of *E. coli* C41(DE3) pLysS cells transformed with pET28b carrying Pr_pa4366-*gfp* (top) or pET28b carrying Pr_pa4366-*gfp* and *dinJ*R13A (bottom).

Table S1. *E. coli* strains and plasmids used in this work

***E. coli* strain**	**Description**	**Source**
XL1-Blue	Cloning and plasmid purification, Tet^R	Stratagene
BL21 (DE3)	Overexpression of recombinant proteins	Invitrogen
C41(DE3) pLysS	In vivo transcription assays, Cam^R	Lucigen
**Plasmid**	**Description**	**Source**
pGEM-T easy	Cloning vector, Amp^R	Promega
pET11b	Expression vector, IPTG inducible, Amp^R	Novagen
pET28b	Expression vector, IPTG inducible, Kan^R	Novagen
pET11b-yafQ_pa4366	pET11b with YafQ ORF from *L. paracasei* 4366	Ferrari et al. 2019
pET28b-yafQ_pa4366	pET28b with YafQ ORF from *L. paracasei* 4366	Maggi et al. 2021
pET11b-yafQ_pa2333	pET11b with YafQ ORF from *L. paracasei* 2333	Ferrari et al. 2019
pET28b-yafQ_pa2333	pET28b with YafQ ORF from *L. paracasei* 2333	This work
pET11b-dinJ_pa4366	pET11b with DinJ ORF from *L. paracasei* 4366	Maggi et al. 2021
pET28b-dinJ_pa4366	pET28b with DinJ ORF from *L. paracasei* 4366	Ferrari et al. 2019
pET28-dinJR13A	pET28b with DinJR13A ORF from *L. paracasei* 4366	This work

Table S2. Oligonucleotides used in this work

Primer	Sequence	Strain	Description
yafQ_pa plus	CATATGTATAGTCTGGTTCCGACG	4366, 2333	YafQ cloning
YafQ_pa minus	GGATCCTATTTACCCAGAAGGTTATGA	4366, 2333	YafQ cloning
dinJ_pa4366 plus	CATATGGCAGCCACAAAGAAAGAA	4366	DinJ cloning
dinJ_pa4366 minus	GGATCCCTATACATTCAAGTCTCTCCAC	4366	DinJ cloning
dinJR13A plus	GAAACTCGCTTGAATATTGCTGTTGATCCGGAATTAAAAAGTGC	4366	Mutagenesis
dinJR13A minus	GCACTTTTTAATTCCGGATCAACAGCAATATTCAAGCGAGTTTC	4366	Mutagenesis
Pr-plus_*DY682*	DY682-AACGTCTGATGGCAGTTACG	4366	EMSA
Pr-minus_*DY682*	DY682-GATCAACACGAATATTCAAGCG	4366	EMSA
LPr-plus_*DY682*	DY682-CGTCCTCAAGCTGCTCTTG	--	EMSA
LPr-plus_*DY682*	DY682-GTTTGCCCAAAGCGCATTGC	--	EMSA
Pr_pa4366 plus	GTGATCGATCTTTGAGGACCG	4366	Pr-gfp cloning
Pr_pa4366 minus	CTCCTTTACTCATTGCTAACCAC	4366	Pr-gfp cloning
gfp plus	GTGGTTAGCAATGAGTAAAGGAG	--	Pr-gfp cloning
gfp minus	TTATTTGTATAGTTCATCCATGCC	--	Pr-gfp cloning
Pr-gfp-BglII plus	TATAGATCTGTGATCGATCTTTGAGGAC	--	Pr-gfp cloning
Pr-gfp-HindIII minus	TATAAGCTTTTATTTGTATAGTTCATCCATGC	--	Pr-gfp cloning

Underlined sequence indicates the overlapping region.
